# Quality of Life of People Living with HIV/AIDS in the Ho Municipality, Ghana: A Cross-Sectional Study

**DOI:** 10.1155/2017/6806951

**Published:** 2017-10-24

**Authors:** James Osei-Yeboah, William K. B. A. Owiredu, Gameli Kwame Norgbe, Sylvester Yao Lokpo, Christian Obirikorang, Emmanuel Alote Allotey, John Gameli Deku, Emmanuel Akomanin Asiamah, Nana Yaw Barimah Manaphraim, Prince Senyo Kwasi Nyamadi, Edward Yiadom Boakye, Tibemponi Ntoni, Roseline Avorkliyah, Romeo Asumbasiya Aduko, Seyram Tetteh Quarshie, Maxwell Jenkins Gbemu

**Affiliations:** ^1^Department of Medical Laboratory Sciences, School of Allied Health Sciences, University of Health and Allied Sciences, Ho, Ghana; ^2^Department of Molecular Medicine, School of Medical Sciences, Kwame Nkrumah University of Science and Technology, Kumasi, Ghana; ^3^Department of Clinical Biochemistry, Diagnostic Directorate, Komfo Anokye Teaching Hospital, Kumasi, Ghana; ^4^School of Allied Health Sciences, University of Health and Allied Sciences, Ho, Ghana; ^5^Health Information Unit, Ho Municipal Hospital, Ghana Health Service, Ho, Volta Region, Ghana; ^6^Laboratory Department, Volta Regional Hospital, Ghana Health Service, Ho, Volta Region, Ghana

## Abstract

Quality of life (QoL) is an important component in the evaluation of the wellbeing of people living with HIV/AIDS (PLHIV). This study was aimed at evaluating the QoL of PLHIV attending the antiretroviral clinics in the Ho municipality. A cross-sectional study was conducted from January 2017 to April 2017 involving 158 purposively selected HIV-positive patients who were attending the antiretroviral clinics both in the Volta Regional Hospital and Ho Municipal Hospital. An Interviewer administered standard questionnaire (WHOQOL-HIV Bref) was used to collect information on sociodemography, medical history, and the quality of life (QoL) of the respondents. Among these 158 HIV-positive respondents, 126 (79.75) and 14 (8.86) presented with excellent and good overall QoL, respectively, whilst 18 (11.39) had their life negatively affected by HIV/AIDS. Religious/personal beliefs (19.62%) were the most affected QoL component, followed by the physical (15.82%) and level of independence (15.19%) domains. Patients' occupation, perception of health, sexual activity, and state of the disease were associated with poor overall QoL. In general, being an HIV-infected man, symptomatic patient, not being sexually active, or being ART naïve was also associated with poorer QoL in several HIV/AIDS QoL domains.

## 1. Introduction

The Human Immunodeficiency virus (HIV) infection and its associated pandemic of Acquired Immune Deficiency Syndrome (AIDS) have burdened the population with serious public health and socioeconomic challenges over the years [1]. The disease does not only affect the patients' physical condition, but also affects their sociocultural relations, mental health, and financial aspects of life [2, 3].

The introduction of antiretroviral treatment (ART) has drastically changed the course of the disease from a rapidly progressive catastrophic illness to a chronic disease with reduction in mortality rate, opportunistic infections, and length of hospitalisation [4, 5]. However the increase in the access to biomedical interventions such as ART for people living with HIV/AIDS (PLHIV), in the developing world, has not been adequately matched with the requisite psychosocial treatments to help improve the effectiveness of biomedical interventions [3]. Optimizing care for PLHIV requires an understanding of the factors that contribute to physical health, psychological wellbeing, social relationships, and quality of life [5].

Quality of life has been considered synonymous with health status, functional status, psychological wellbeing, happiness with life, satisfaction of needs, and assessment of one's own life [6]. Assessment of quality of life has become an important outcome measure in the management of HIV/AIDS and reflects improvement or otherwise of the health experience and satisfaction with care among patients living with HIV/AIDS [7, 8].

In Ghana, the Volta region with an HIV prevalence of 2.7% higher than the national prevalence of 2.4% is one of the top HIV regions according to the 2016 national sentinel survey report [9]. According to Folasire et al. [10], QoL assessment helps in making judgments about areas of need and the planning of interventions in the management of PLHIV. A review of the Ghanaian literature shows no available data on QoL of PLHIV in the regional jurisdiction of Volta, the site of the current study. To address this knowledge gap, this study was aimed at assessing the QoL of PLHIV, in the Ho municipality in the Volta region of Ghana, using the World Health Organization instrument for quality of life, the brief version (WHOQoL-Bref).

## 2. Methods

### 2.1. Study Design and Study Site

A hospital-based cross-sectional study was conducted from January 2017 to April 2017 involving one hundred and fifty-eight (158) purposively selected HIV-positive patients, who were attending the Antiretroviral Clinics at the Volta Regional Hospital (VRH) and the Ho Municipal Hospital. The study participants were selected from a pool of patients 18 to 70 years old, who live in the Ho municipality.

### 2.2. Sample Size Determination

Using the average monthly attendance of HIV/AIDS patients of sixty-five (65) for two previous months (November 2016 and December 2016), a total study population of 260 was generated for the four-month study duration. Using the Raosoft online sample size calculator (http://www.raosoft.com/samplesize.html), the recommended minimum sample of 156 participants was calculated at 95% confidence level, 5% margin of error, and a response distribution of 50%.

### 2.3. Instrument for Data

A questionnaire, consisting of sociodemographic, medical, and the WHOQOL-HIV Bref instrument was administered at the time of the interview to assess each study participant.

The questionnaire consisted of 31 items in six (6) domains (physical health, psychological health, level of independence, social relationships, environment, and religious/personal beliefs, as well as one item each for overall quality of life and general health perception). The physical health domain measured pain and discomfort, energy and fatigue, and sleep and rest. The psychological domain measured positive feelings, thinking, learning, memory and concentration, self-esteem, bodily image and appearance, and negative feelings. The level of independence domain measured mobility, activities of daily living, dependence on medications or treatments, and work capacity. The social relationships domain measured personal relationships, social support, and sexual activity. The environmental domain measured physical safety and security, home environment, financial resources, health and social care: accessibility and quality, opportunities for acquiring new information and skills, participation in and opportunities for recreation and leisure activities, and physical environment (pollution, noise, traffic, climate, and transport). The religious/personal beliefs domain measured forgiveness and blame, concerns about the future, and death and dying [11]. Each item contained a 5-point Likert-type scale that best represented their opinion, based on their life over the previous 4 weeks. On the scale, one (1) indicated low and negative perceptions, whilst five (5) indicated high and positive perceptions, which denoted better QoL. Negatively worded items were reverse scored, and all scores were checked for appropriate range (between (1) and (5)).

### 2.4. Data Analysis

The percentage scores were calculated as the sum of individual scores obtained in a domain divided by the total attainable score in that domain multiplied by 100. Thus the percentage scores range from a minimum of 25 to a maximum of 100 [12]. Quality of life scores were categorized into three (3) sections; with scores ≥ 80 denoting excellent QoL, 60 to 79 denoting a good QoL, and <60 representing poor QoL [13]. Statistical analysis was performed using the Statistical Package for Social Science (SPSS) software package, version 21.0. Data were presented using frequency and percentages, median and corresponding interquartile range in parenthesis for descriptive variables, and testing between proportions were carried out using Fisher Exact and chi square test where appropriate, whilst Independent Sample Mann–Whitney *U* test was used to compare two medians. At all times, *p* < 0.05 was considered as statistically significant.

### 2.5. Ethical Consideration

Informed consent was obtained from all participants after the study procedure was clearly explained to them in a language they understand (either English or Ewe). Approval for the study was obtained from the facilities at both the Volta Regional Hospital (VRH), Ho, and the Ho Municipal Hospital. Ethical Clearance for the study was granted by the Ethical Review Committee of the School of Allied Health Sciences of the University of Health and Allied Sciences, Ho* (UHAS-SAHS-ERSC:026A/2017)*. No patient was denied the appropriate care for declining to participate in the study.

## 3. Results

The study population consisted of 158 patients, 88 (55.70%) from the Volta Regional Hospital and 70 (44.30%) from the Ho Municipal Hospital. The male population represented 42 (26.58%) and the female 116 (73.42%) of patients from the two facilities. Majority of the participants 104 (65.82%) had primary school education and 62 (39.24%) were married as at the time of data collection. One hundred twenty-five (79.11%) participants were employed in the informal sector and 10.76% of participants had no gainful employment. Majority of the participants were in the asymptomatic stage of the disease 126 (79.75%) and a total of 101 (63.92%) patients were on ART (See Table 1).

The overall quality of life median percentage score and interquartile range among the study respondents were 71.29 and 9.31, respectively. The psychological domain recorded the lowest median percentage score (68.00) and highest component score was observed in the social relationship domain (75.00). Though not statistically significant, the median scores of the overall QoL, the physical and social relationship, and spiritual/religion/personal beliefs domains were higher for the females compared to their male counterparts (See Table 2).

Among the study population, 126 (79.75) were graded as presenting with an excellent overall QoL, 14 (8.86) presented with good overall QoL, and 18 (11.39) had their life negatively affected by the disease. On the question of health satisfaction of PLHIV, 123 (77.85%) appraised their health as excellent, 11 (6.96%) assessed their health as good, and 24 (15.19%) appraised their health as poor. Religious/personal beliefs domain which looks at the patients' inclination to forgiveness and blame and concerns about the future, death, and dying recorded the poorest score 31 (19.62%), followed by physical component (pain and discomfort, sleep and rest, mobility, activities of daily living, and work capacity), 25 (15.82%), and level of independence 24 (15.19%) (See Table 3).

As seen from Table 4, the respondents' facility of care, gender, educational background, marital status, and antiretroviral therapy had no significant association with the quality of life. Participants who were not gainfully employed presented with the highest affected quality of life. Patients who perceived themselves as having poor health significantly presented with a lower quality of life 18 (20.45) compared to their counterparts who perceived themselves as healthy 0 (0.00%). Significantly poorer QoL was observed among patients who were sexually inactive 15 (18.52) and symptomatic HIV/AIDS patients 8 (25.00) (See Table 4).

A significant gender variation of psychological quality of life among the study population was observed. The male population presented with a poorer psychological QoL (23.81%) than their female counterparts (9.48%). Non-sexually active HIV/AIDS patients had a significantly poorer quality of life (19.75%) compared to sexually active patients (6.49%) for the psychological domain. Patients seeking care at the Ho Municipal Hospital had better quality of life compared to those at the Volta Regional Hospital the psychological quality of life domain and general health perceptions. Symptomatic HIV/AIDS patients had a poorer quality of life (31.25% and 21.88%) as compared to the asymptomatic patients (11.11% and 14.29%) for general health perceptions and physical quality of life domains, respectively. However asymptomatic HIV/AIDS patients had a poorer score (22.22%), compared to the symptomatic patients (9.38%), under the spiritual/religion/personal beliefs. Patients who were not on ART had a significantly poorer quality of life (29.82%) as against patients on ART (13.86%) for spirituality/religiosity/personal beliefs QoL domain (See Table 5).

## 4. Discussion

Quality of life (QoL) refers to the degree of excellence in a person's life at any given period that contributes to satisfaction and happiness of the person and benefits society [14]. The current study aimed to assess the quality of life of people living with HIV/AIDS in the Ho Municipality of Ghana. The demographic profile of the participants showed that majority (73.42%) of the study participants were females. Gender vulnerability to HIV infection is tilted towards females in the Ghanaian and African society [1, 15]. Furthermore, most of the respondents (74.68%) had not attained secondary education at the time of the study. This finding compares with the demographic characteristics of the HIV/AIDS population in the work of S. I. Bello, and I. K. Bello [1] in Nigeria where formal education levels were low among PLHIV.

In the current study, the overall median percentage score of QoL was 71.29, which is lower than reported by Acharya [16] and Giri et al. [17] (80.00) among Nepalese PLHIV, but higher than reported by Oliveira et al. [18], (62.5) among Brazilian PLHIV. Other works in the literature in the African setting presented their QoL scores in means, therefore making direct comparison of scores with the current study not plausible [1, 6, 10]. With the exception of the psychological domain in the Brazilian study [18], all the component QoL percentage median scores recorded in this study were found to be higher than that recorded by the previous cited works.

The social and environment domains which are a reflection of the physical environment, poor living conditions, and how PLHIV are affected by societal discrimination and stigmatization, as well as HIV/AIDS' influence on patients' sexual desire, personal relationships, and family life, could be said to be better in Ho municipality than in other jurisdictions [1, 6, 16–18]. This may be due to effective social support network and reduced exposure to discrimination and stigmatization [10].

In this study population, HIV/AIDS negatively affected the quality of life of 11.39%, with the most affected domain being the spiritual/religious/personal belief domains (19.62%). The least affected domain was the environmental domain (5.70%), which measured physical safety and security, home environment, financial resources, health and social care, accessibility and quality, opportunities for acquiring new information and skills, participation in and opportunities for recreation and leisure activities, and physical environment [19].

Though not statistically significant, the study observed that, with the exception of one, all the patients who had attained tertiary level education at the time of the study presented with excellent quality of life (Table 4). According to Liping et al. [20] who also reported a similar finding in an earlier work, the reason may be that people with higher educational attainment have a more enlightened attitude towards the disease with the increasing public awareness of HIV. Patients who are more educated can better understand the disease state and the instructions given on drug usage, which invariably enhances their QoL [1].

The symptomatic patients significantly presented with a lower overall quality of life, lower physical QoL, and lower general perception of health but had better spirituality/religiosity/personal beliefs and this agrees with the findings of Folasire et al. [10] in a study conducted in Nigeria. Accounting for high QoL score in the spirituality/religiosity/personal beliefs domain among PLHIV in Northern Ethiopia, Tesfay et al. [21] posited that people tend to be spiritual and religious when confronted with issues that are beyond them; they engage in spiritual and religious reflections, treasuring the gifts in their lives, accepting and surrendering to the approach of their death. This state of heightened spirituality reflects on the high score on the spirituality/religiosity/personal beliefs domain.

According to Shan et al. [22], among medical history variables, HIV-infected persons with worse health conditions are inclined to have worse QoL. One of the most acknowledged clinical variables associated with QoL of PLHIV is the breakdown of immunity which is the main cause of symptoms in HIV and is associated with lower QoL [20, 22].

Contrary to the findings of Manhas [19] who attributed the lower quality of life among women living with HIV/AIDS in India to their male counterpart as a reflection of a patriarchal society where gender inequality leads to higher discrimination, stigmatization, and abuse of a female living with HIV/AIDS, in the current study the male participants presented with a poorer psychological quality of life (Table 5). On the other hand Folasire et al. [10] did not find any significant difference in QoL scores between males and females living with HIV/AIDS in Nigeria.

As mentioned by other reports, QoL is associated with employment and income status [3, 22, 23]; this study found that patients who were not gainfully employed presented with a significantly lower QoL, (41.18%), which was more than five times when compared with those who were employed in the informal sector and more than thrice those employed in the formal sector (*p* = 0.0017) (Table 4).

Patients' self-appraisal of their health significantly predicted their quality of life, with lower QoL recorded among those who perceived themselves as ill. The significantly better quality of life in the psychological and health perception among patients attending ART clinic at the Municipal hospital compared to those obtaining care from the regional hospital may require further studies to establish the causal relationship. However, some studies have alluded to the impact of health professionals on the wellbeing of patients presenting with chronic diseases such as PLHIV through comprehensive and consistent counseling of patients on antiretroviral drugs and education on their disease state [1].

## 5. Conclusion

In general, being an HIV-infected man, symptomatic patient, not being sexually active, or being ART naïve was linked to poorer QoL in several HIV/AIDS QoL domains. Furthermore, where PLHIV's receive care also affects their QoL.

Our findings suggest that patient-reported measures of health status and related concepts may help provide a feasible, reliable, and valid method to assess the impact of HIV/AIDS and future management interventions to improve patient outcomes.

## Figures and Tables

**Table 1:** Sociodemographic characteristics of people living with HIV/AIDS in Ho municipality.

Parameter	Frequency	Percentage
Total	158	100
Facility		
Regional Hospital	88	55.70
Municipal Hospital	70	44.30
Gender		
Male	42	26.58
Female	116	73.42
Educational background		
No education	14	8.86
Primary	104	65.82
Secondary	30	18.99
Tertiary	10	6.33
Marital status		
Single	85	53.80
Cohabitation	11	6.96
Married	62	39.24
Occupation status		
Formal	16	10.13
Informal	125	79.11
Not employed	17	10.76
Stage of disease		
Asymptomatic	126	79.75
Symptomatic	32	20.25
Antiretroviral therapy		
ART	101	63.92
ART naïve	57	36.08

Data is presented as frequency with corresponding percentage.

**Table 2:** Quality of life score and component quality of life scores stratified by gender.

Parameter	Total (158)	Female (116)	Male (42)	*p* value
Overall QoL	71.29 (9.31)	71.63 (8.23)	69.42 (10.15)	0.167
Physical	70.00 (25.00)	75.00 (25.00)	70.00 (20.00)	0.292
Psychological	68.00 (12.00)	68.00 (12.00)	68.00 (13.00)	0.071
Level of independence	70.00 (15.00)	70.00 (15.00)	70.00 (16.25)	0.080
Social relationships	75.00 (15.00)	75.00 (15.00)	70.00 (20.00)	0.239
Environment	72.50 (12.50)	72.50 (12.50)	72.50 (12.50)	0.777
Spiritual/religion/personal beliefs	70.00 (25.00)	70.00 (25.00)	67.50 (21.25)	0.635

Data is presented as median (interquartile range). *p* is significant at 0.05.

**Table 3:** Components of the quality of life of people living with HIV/AIDS in the Ho municipality.

Parameter	Excellent	Good	Poor
Overall QoL	126 (79.75)	14 (8.86)	18 (11.39)
Health satisfaction	123 (77.85)	11 (6.96)	24 (15.19)
Physical	56 (35.44)	77 (48.73)	25 (15.82)
Psychological	33 (20.89)	104 (65.82)	21 (13.29)
Level of independence	40 (25.32)	94 (59.49)	24 (15.19)
Social relationships	51 (32.28)	89 (56.33)	18 (11.39)
Environment	26 (16.45)	123 (77.85)	9 (5.70)
Spiritual/religion/personal beliefs	62 (39.24)	65 (41.14)	31 (19.62)

Data is presented as frequency with corresponding percentage in parenthesis.

**Table 4:** Variables associated with the overall Quality of Life of People Living with HIV and AIDS in the Ho municipality.

Parameter	Excellent	Good	Poor	*p* value
Facility				
Regional Hospital	68 (77.27)	7 (7.96)	13 (14.77)	0.3121
Municipal Hospital	58 (82.86)	7 (10.00)	5 (7.14)	
Gender				
Male	31 (73.81)	5 (11.90)	6 (14.29)	0.5295
Female	95 (81.90)	9 (7.76)	12 (10.34)	
Educational Background				
None	9 (64.29)	4 (28.57)	1 (7.14)	0.2201
Basic	84 (80.77)	8 (7.69)	12 (11.54)	
Secondary	24 (80.00)	2 (6.67)	4 (13.33)	
Tertiary	9 (90.00)	0 (0.00)	1 (10.00)	
Marital status				
Single	69 (81.18)	7 (8.23)	9 (10.59)	0.9547
Cohabitation	8 (72.73)	1 (9.09)	2 (18.18)	
Married	49 (79.03)	6 (9.68)	7 (11.29)	
Occupational status				
Formal	13 (81.25)	1 (6.25)	2 (12.50)	0.0017
Informal	104 (83.20)	12 (9.60)	9 (7.20)	
Not employed	9 (52.94)	1 (5.88)	7 (41.18)	
Antiretroviral Therapy				
ART	82 (81.19)	10 (9.90)	9 (8.91)	0.3813
ART Naive	44 (77.19)	4 (7.02)	9 (15.79)	
Perception of Health				
Ill health	62 (70.45)	8 (9.09)	18 (20.45)	0.0003
Healthy	64 (91.43)	6 (8.57)	0 (0.00)	
Sexual activity				
Active	66 (85.71)	8 (10.39)	3 (3.90)	0.0144
Nonactive	60 (74.07)	6 (7.41)	15 (18.52)	
Disease state				
Symptomatic	22 (68.75)	2 (6.25)	8 (25.00)	0.0242
Asymptomatic	104 (82.54)	12 (9.52)	10 (7.94)	

Data is presented as frequency with corresponding percentage in parenthesis. *p* is significant at 0.005.

**Table 5:** Associated variable with the component of quality of life of people living with HIV/AIDS in the Ho municipality.

QoL Domain	Parameter	Excellent	Good	Poor	*p* value
Psychological quality of life					
Gender	Male	5 (11.90)	27 (64.29)	10 (23.81)	0.0303
	Female	28 (24.14)	77 (66.38)	11 (9.48)	
Sexual activity	Active	18 (23.38)	54 (70.13)	5 (6.49)	0.0476
	Non Active	15 (18.52)	50 (61.73)	16 (19.75)	
Facility	Regional	11 (12.50)	63 (71.59)	14 (15.91)	0.0128
	Municipal	22 (31.43)	41 (58.57)	7 (10.00)	
Spiritual/religion/personal beliefs					
Disease state	Symptomatic	21 (65.63)	8 (25.00)	3 (9.38)	0.0027
	Asymptomatic	41 (32.54)	57 (45.24)	28 (22.22)	
Therapy	ART	44 (43.56)	43 (42.57)	14 (13.86)	0.0449
	Naive	18 (31.58)	22 (38.60)	17 (29.82)	
General health perceptions					
Disease state	Symptomatic	20 (62.50)	2 (6.25)	10 (31.25)	0.0178
	Asymptomatic	103 (81.75)	9 (7.14)	14 (11.11)	
Facility	Regional	65 (73.86)	4 (4.55)	19 (21.59)	0.0244
	Municipal	58 (82.86)	7 (10.00)	5 (7.14)	
Physical quality of life					
Disease state	Symptomatic	5 (15.63)	20 (62.50)	7 (21.88)	0.0312
	Asymptomatic	51 (40.48)	57 (45.24)	18 (14.29)	

Data is presented as frequency with corresponding percentage in parenthesis. *p* is significant at 0.05.
